# Somali refugees in urban neighborhoods: an eco-social study of mental health and wellbeing

**DOI:** 10.3389/fpsyt.2024.1307509

**Published:** 2024-02-19

**Authors:** Guntars Ermansons, Hanna Kienzler, Peter Schofield

**Affiliations:** ^1^ Department of Global Health & Social Medicine, Faculty of Social Science & Public Policy, King’s College London, London, United Kingdom; ^2^ Department of Population Health Sciences, School of Life Course & Population Sciences, King’s College London, London, United Kingdom

**Keywords:** refugees, mental health, wellbeing, eco-social niches, urban neighborhoods

## Abstract

**Background:**

Impact of pre-migration trauma and post-migration settlement on refugee mental health and wellbeing is well-documented. However, little research has focused on the specific places where refugees settle and spend their daily lives within the post-migration context. This study adopts an eco-social perspective to explore the relationship between urban neighborhoods and refugee mental health and wellbeing.

**Methods:**

We conducted twenty-six qualitative interviews with Somali refugees in London and Bristol in the UK. The transcripts were coded using an inductive approach and analyzed through thematic analysis.

**Results:**

Somali refugees navigate a complex urban environment comprising various neighborhood features which include important places near home, interactions with neighbors, and community spaces. While these features afford them resources to improve mental health and wellbeing, they also present challenges such as high urban density, exposure to violence or discrimination, and neighborhood disorder.

**Conclusion:**

The societal and physical features of urban neighborhoods intersect with refugee experiences of adversity, trauma and stress over time. As eco-social niches, urban neighborhoods are both accommodating, safe and familiar, as well as alien, threatening and unwelcoming. To support mental health and wellbeing and ensure successful settlement, it is essential to recognize the agency of refugees and provide continuous support throughout the entire asylum process and after, ensuring stable and safe living conditions.

## Introduction

Within the asylum landscape, cities have emerged as significant sites of refugee destination and settlement (1). More than 60% of the world’s refugees and asylum seekers live in urban areas and this percentage is probably even higher in Global North countries (2, 3). Literature on ‘urban refugees’ focuses mostly on the Global South^1^ where most refugees are displaced and settled (5–7). Conversely, while there has been a focus on refugee mental health in Global North countries, relatively little is known about the urban experience of refugees and how they navigate their daily lives within urban neighborhoods, particularly understanding how this affects their mental health and wellbeing (8–10).

Our qualitative study contributes to this nascent field by exploring the post-migration experiences of Somali refugees and their mental health and wellbeing in the context of urban neighborhoods in London and Bristol. The Somali refugee community presents an interesting case as they have resided in the UK for a significant period since the outbreak of the Somali Civil War in 1991. Indeed, the Somali population in the UK is the largest in Europe, with an estimated 108,921 Somali-born permanent residents, approximately 59,000 of which have obtained British citizenship (11). A significant number of Somalis living in the UK are also citizens of other EU countries. Their longer-term experience living in UK urban neighborhoods provides crucial insight into how societal and physical features of environment intersect over time with adversity, trauma and stress to shape their mental health and wellbeing.

In our study, we asked the following questions: What urban neighborhood features do Somali refugees consider important for their experiences of settlement and belonging? How do these features shape their mental health and sense of wellbeing? What are the underlying processes that mediate between social and physical environments of neighborhoods and refugee mental health and wellbeing? To answer these questions, we draw on an eco-social framework (12–14) and explore the complexities of the interplay between individual, social, physical and temporal dimensions of everyday refugee life in the city. Research among refugees has underscored that refugee mental health is not only an outcome of individual pre-migration experiences but also the result of complex interactions between people and their post-migration environments, both social (e.g., poverty, unemployment, legal challenges, interpersonal violence, social support) and physical (e.g., neighborhood characteristics, green spaces, housing conditions) (12, 15). In other words, our emphasis is on ‘social ecology’ where mental health outcomes experienced by refugees are located within their respective living environments. Everyday stressors and sources of resilience are embedded within such material conditions, social networks, community structures, and the policies of the host society (13). The eco-social framework further posits that “humans create and inhabit, shape and are shaped by their ‘ecological niches’” (14:3), foregrounding the need to recognize refugee agency as a key element in the processes of adaptation and settlement in new environments. This leads to our focus on urban neighborhoods as eco-social niches that play a pivotal role in the experiences of displacement and migration. Understanding urban neighborhoods as eco-social niches enables us to examine how refugees navigate and inhabit urban places on different scales, from housing estates to the city streets, and how making a place for themselves shapes their mental health and wellbeing over time.

In the following, we introduce the UK asylum context, outline a brief history of Somali refugees in the UK and explore some of their conceptions of mental health and wellbeing based on available literature. In the results section, we present three salient themes that emerged from thematic analysis of our data, revealing features of urban neighborhoods as human ecological niches that afford and impede settlement with implications for mental health and wellbeing. We conclude by discussing the implications of our findings for understanding refugee mental health and wellbeing in urban contexts.

### UK asylum landscape and urban refugees

Refugees in the UK are increasingly facing restrictive asylum regimes embedded within broader ‘hostile environment’ policies (16). For many refugees, their very first experience as asylum seekers in the UK is that of languishing in unfavorable and unpredictable environments which severely limit the “individual and collective process of creating spaces of belonging” (17). For instance, around 93% of asylum seekers are dispersed to housing estates which are often located in areas of economic decline where refugees are exposed to social isolation and discrimination which elevate their mental health and wellbeing risks (18–20).

Due to long delays in processing asylum claims in the UK, asylum seekers are forced to live in limbo for months on end with an average waiting time of between one and three years (21). Once individuals receive refugee status, they undergo a 28-day transition period^2^ from asylum to welfare system, which creates financial instability and upheaval due to frequent changes in accommodation (23, 24) and can have detrimental effects on the mental health of refugees (25). Many refugees live in poverty and face unemployment, underemployment, inadequate housing conditions, limited support networks, low English proficiency and discrimination which, in turn, are variously experienced and exacerbated by additional intersectional factors such as LGBTQ+ identity, gender, age and disability (26–28).

To positively facilitate refugee settlement in urban neighborhoods, ‘refugee-integration-opportunity structures’ (29) and inclusive social and physical environments have been shown to be essential (30, 31). Specific locations within cities, such as public parks, libraries and urban allotments, can offer therapeutic sensory and embodied experiences that improve mental health and a sense of belonging for refugees (31–34). Voluntary onward migration also plays a significant role as refugees seek to resettle in urban neighborhoods that provide better opportunities and closer proximity to their family and community networks (35, 36). These findings appear to be in line with the effect of ethnic density on better mental health outcomes and reduced risk of psychosis in migrants (37). However, for refugee communities to thrive, the broader cultural and ethnic diversity of the city is equally crucial in promoting acceptance and reducing hostility (38).

### Somali refugees in the UK

Somali settlement in the UK dates to the late nineteenth century, with seamen from colonial British Somaliland settling in port cities like London, Cardiff and Liverpool. Later in the mid-twentieth century, Somali migrants arrived in industrial cities such as Sheffield, Manchester and Birmingham (39). However, the demographic shifted notably after the full-scale outbreak of the Somali Civil War in the early 1990s, leading to the arrival of refugees who faced challenges such as limited employment opportunities, language barriers and reliance on the welfare system (40, 41). Today, there are several London boroughs with a notable Somali community presence (42), and Somalis are the largest ethnic minority group in Bristol according to the City Council (2022). In both cities, Somalis live in some of the most deprived areas, characterized by high unemployment, poverty and poor housing (43, 44). Up to 75% of UK Somali refugees are unemployed (45), and they are among the most affected ethnic minority groups suffering from poor health conditions such as tuberculosis (46).

Such post-migration factors have been found to contribute to an increased risk of poor mental health outcomes in Somali refugees (47–49). For example, a comparative study of Somali refugees in Minneapolis, USA, and London, UK, found refugees in both cities felt disempowered and disillusioned due to restrictive social and legal environments, limiting their agency and socio-economic participation (50). Somali refugees in London experienced additional challenges relating to family separation, legal uncertainties and unemployment compared to those in Minneapolis, and they were more likely to report major depression and any mental disorder. Another study showed that Somali refugees in London moved an average of four times in five years before securing permanent accommodation. This resulted in considerable stress and negative impacts on their lives, such as family and social ties, child development and stable access to health and social care services (51). Somali refugees who relocated within five years of arriving in the UK were more vulnerable to psychiatric disorders (25).

While little is known about prevalence rates of mental disorders, an earlier study by Bhui and colleagues (52; see also 53) found that more than one-third of Somali refugees surveyed via GP registers and community sites in the UK had a mental disorder, the most prevalent being common mental disorders and post-traumatic stress disorder. Such mental health problems are further increased due to intra-community stigma surrounding mental health issues and limited culturally appropriate mental health services (54–56). Such services need to take Somali conceptions of mental health into account which intertwine social and spiritual wellbeing, embedded within collective experiences rather than individualized concerns (57, 58). For instance, Somalis commonly use idioms of distress such as ‘thinking too much’ to draw attention to social ruptures, injustice and practical problems like social isolation, poverty and racism. They may also present with physical symptoms such as headaches, insomnia and chest pain (59–61). Family members are often the first to address distress, with faith-based organizations and cultural activities providing support, a sense of community and spiritual guidance (62, 63). This clearly shows that the social ecology in which Somalis live is not only implicated in their mental health and wellbeing, but also in the ways mental health care and social support are provided.

## Methodology

This study followed a qualitative approach to investigate how features of urban neighborhoods afford and impede settlement with implications for the mental health and wellbeing of Somali refugees. To ensure rigor and cultural sensitivity, an advisory group was formed consisting of six Somali community representatives and an academic with extensive experience of working on mental health issues in post-conflict societies. The advisory group provided feedback on the early stages of the study and on the development of the interview guide.

Between September 2020 and April 2022, semi-structured interviews were carried out at three sites, South and Northwest London and Bristol, with substantial Somali diaspora communities allowing comparisons between them. Although in both cities participants lived in some of the most deprived urban neighborhoods in the UK, they also provided different contexts in terms of population size, urban density and community spaces. Research in South London took place in Brixton, an inner-city neighborhood characterized by comparatively greater urban density than the northwest neighborhood of Willesden and its more suburban surrounding areas. Interviews conducted in Bristol took place in the inner-city area of Easton. Brixton and Willesden are amongst the 20% most deprived neighborhoods in the country and Easton is amongst the worst 10% (Indices of Deprivation 2015 and 2019).

A total of twenty-six Somali refugees took part in the study (**Table 1**). Eighteen Somali refugees were living in London (eight in the Northwest and ten in South London) and eight in Bristol. Participants comprised eleven women and fifteen men between the ages of twenty and sixty-five; six participants disclosed a diagnosis of mental health disorder, all of whom were living in London. Most participants had lived in the UK for an average of fifteen years, with the longest period being thirty-one years and the shortest being five. Participants were recruited in close collaboration with two voluntary sector organizations specializing in community outreach and psychosocial support for Somali refugees. A purposeful sampling approach was used to recruit participants from the Somali diaspora in the two cities, allowing us to find cases that were ‘information rich’ (64). We sought to include female and male participants who currently lived in London and Bristol, although we did not specify the length of residence in the inclusion criteria. Specifically, participants were included if they were Somali born (settled or re-settled in UK as a refugee); were current UK residents; have refugee status (indefinite leave to remain) in UK or other European country; may or may not have gained British or other European citizenship; were eighteen – sixty-five years old; were able to give informed consent.

**Table 1:** Study participants*.

Participant	Gender	Age	Marital status	Employment status	Arrived in UK
BF1	F	40-50	Married	Unemployed	2005-2010
BF2	F	40-50	Divorced	Unemployed	2015-2020
BM1	M	20-30	Married	Employed	2005-2010
BM2	M	40-50	Married	Employed	2000-2005
BM3	M	40-50	Married	Self-employed	2000-2005
BM4	M	20-30	Single	In education	2015-2020
BM5	M	20-30	Single	Unemployed	1990-1995
BM6	M	60-70	Married	Retired	1990-1995
LF1	F	40-50	Married	Unemployed	2010-2015
LF2	F	40-50	Single	Part-time	2000-2005
LF3	F	40-50	Married	Unemployed	2015-2020
LF4	F	30-40	Married	Unemployed	2005-2010
LF5	F	30-40	Married	Volunteering	2000-2005
LF6	F	40-50	Divorced	Unemployed	2000-2005
LM1	M	40-50	Divorced	Unemployed	2005-2010
LM2	M	30-40	Single	Unemployed	2005-2010
LM3	M	40-50	Single	Unemployed	2010-2015
LM4	M	40-50	Married	Employed	1990-1995
BrF1	F	40-50	Married	Self-employed	2000-2005
BrF2	F	40-50	Divorced	Self-employed	2000-2005
BrF3	F	40-50	Widowed	Self-employed	2010-2015
BrM1	M	50-60	Married	Self-employed	1990-1995
BrM2	M	40-50	Divorced	Employed	2010-2015
BrM3	M	40-50	Married	Employed	2005-2010
BrM4	M	30-40	Married	Self-employed	2010-2015
BrM5	M	30-40	Divorced	Self-employed	2010-2015

*BF, female/Northwest London; BM, male/Northwest London; LF, female/South London; LM, male/South London; BrF, female/Bristol; BrM, male/Bristol. To protect participant anonymity, the age and year of arrival in the UK is provided within the 10-year and 5-year range, respectively.

A semi-structured interview guide was developed to focus on the neighborhood and place-specific factors and their relevance for refugee mental health and wellbeing. Most of the interviews occurred face-to-face in the office of one of the Somali community organizations in Northwest London and in public spaces, such as Somali cafés. One interview was conducted online, one via telephone, and two at the participants’ homes. Most interviews took place in English and, for the participants who switched between English and Somali, Somali representatives from the community organizations provided a translation. Interviews ranged from thirty to ninety minutes. All interviews were audio recorded for transcription and analysis.

Interviews were conducted by the first author who has previous experience of conducting research with Somali diaspora. All participants received detailed information about the study prior to their interview. We made it clear that the interviews would focus on how their current living environments and post-migration experiences affected their mental health and wellbeing, rather than delving into potentially traumatic pre-migration events. Informed consent was obtained before each interview, either in writing for face-to-face interactions or verbally for interviews conducted over the phone or internet. For those hesitant to sign documents, verbal consent was deemed acceptable. This approach is often necessary in refugee research, where past persecution and a current status of vulnerability and marginalization heighten concerns about confidentiality, anonymity, and trust (65, 66).

Interviews were thematically analyzed (67) through an initial open coding process aided by NVivo 12. This was followed by a focused coding to draw out categories and key themes from the data. The coded data were organized into categories, establishing connections between these categories and their properties, including settlement experiences, social and material living conditions, daily life routines, principal stressors, and sources of support. This interpretative process enabled the identification of three distinct but interconnected themes (**Table 2**) which are further elucidated in the subsequent results section: 1) Local Area: Settling and Finding Safety; 2) Neighbors and Everyday Social Integration; and 3) Life and Wellbeing in Urban Communities.

**Table 2:** Themes and sub-themes.

Theme	Sub-theme	Description
**Local Area: Settling and Finding Safety**	Familiarity and Attachment	The process of becoming familiar with and attached to local amenities which facilitates a sense of home in a new environment.​ Challenges faced by refugees when first arriving in a new country, including feelings of isolation, disorientation and search for safety.
	Spatial Routines and Therapeutic Significance	The importance of developing spatial routines that provide comfort and a sense of safety, and the therapeutic significance of certain locations and places within the neighborhood.
	Navigating Urban Environments	Challenges related to moving through busy urban landscapes, like public transportation systems. The stresses associated with living in densely populated areas, dealing with noise and air pollution, and the overall nature of urban life.
**Neighbors and Everyday Social Integration**	Initial Relationships and Support	The role of neighbors, both Somali and non-Somali, in helping refugees settle and integrate into the host society, including language support, socialization and forming close-knit relationships​.
	Reciprocity and Cultural Exchange	Everyday interactions and mutual support between refugees and their neighbors, fostering social integration and a sense of belonging​ and place attachment.
	Incidents and Safety Concerns	Challenges and stressors faced by refugees living in shared accommodation. Encounters with anti-social behavior and concerns about racism and neighbourhood violence in certain areas and housing environments.
**Life and Wellbeing in Urban Communities**	Somali Diaspora and Social Networks	The influence of Somali diaspora on local economies and the establishment of community spaces, which facilitated friendships, social ties, and a sense of belonging and agency​.
	Community Beyond Somali Diaspora	Engagement with wider community groups and centres, particularly for those facing mental health issues or intra-community stigma, and the formation of supportive networks outside the Somali community.

## Results

Our findings show that Somali refugees navigate a complex urban environment comprising various neighborhood features which include important places near home, interaction with neighbors, and community spaces. While these features afford them resources to improve mental health and wellbeing, they also present challenges such as high urban density, exposure to violence or discrimination, and neighborhood disorder. Our framework reflects the notion of neighborhood as an eco-social niche embedded within the broader urban context and the themes represent overlapping zones of living.

### Local area: settling and finding safety

All participants emphasized the significance of the local area in their settlement experience and wellbeing. They used the term *xaafad* (neighborhood) to refer to the neighborhood area in proximity to their homes consisting of places they sought to access after arriving, such as shops, parks, mosques, schools, cafés, pharmacies and markets. While participants did not strictly define the boundaries of such local areas, they described them as a network of points of interest within a walking distance or with a short ride between them. Daily access to these places helped participants become familiar with their new environment, developing a sense of attachment and safety over time. This was particularly significant during the early periods of settlement in the UK and when moving and settling in a new neighborhood.

Arriving in a new environment was initially an isolating and disorienting experience for most participants. A middle-aged married woman and mother of two remembered this first experience as follows: “*When I came to this country, I felt depressed because I ran away from conflict. I did not know anyone, and I felt isolated. I did not know anyone to talk to … Now, I feel like I’m home because I get used to living here, yeah, and I’m familiar with the area, so I feel like I’m home*” (LF5). Arriving from Mogadishu in 2004, she first lived in Croydon, South London, in temporary accommodation where she felt isolated and lonely. She described her primary concern to get to a ‘safe place’ and ‘away from conflict’, but once arrived: “*not knowing anyone as a very young person, it was quite a scary time for me, yeah*”. When she moved to Brixton in South London, she established connections with other Somalis there, enrolled in college and now volunteers to help Somalis from the neighborhood and surrounding areas.

For all participants, time played a crucial role in settling in. Having regular access to places of interest in the vicinity contributed to increasing familiarity and eventually led to the formation of a sense of attachment to place that many described as ‘feeling home’. However, most participants had moved several times within and between cities before settling in their current address. A middle-aged male participant in Bristol who had previously lived in London and Birmingham, explained motivations for him to move were connected to having friends and employment opportunities. However, he was also drawn to Bristol due to its smaller size. He could easily access his workplace, hospital, pharmacy, market and city centre which were within a ten-minute walk from his home in the Somali-populated neighborhood of Easton.

Familiarity with the local area also afforded access to locations with therapeutic significance for some participants. A middle-aged man who lived in supported housing in Brixton highlighted the importance of going out and ‘having some fun’ to help him cope with feeling depressed and stressed. He noted: “*I like it because it’s a nice place, the market, my friends are here, men’s [mental health support] group*” (LM2). Similarly, another participant emphasized the significance of her local area in managing her mental health condition, particularly when her social anxiety worsened and prevented her from traveling further away. Her routine mostly involved going to the park and sitting down by herself, which she found to be soothing. These examples illustrate that people developed spatial routines that enhanced their sense of familiarity, attachment and safety, and connected to this, feelings of wellbeing.

Study participants valued such spatial routines and expressed a preference for maintaining a sense of safety and attachment to their familiar surroundings, even if it meant living in substandard housing or densely populated areas. For example, a single mother residing in a hostel in Brixton for two-and-a-half years faced challenges in securing council housing in the same area but was determined to remain there. Despite feeling unsafe and stressed within the hostel, she emphasized the importance of proximity to her workplace, amenities and her son’s school. She stated, “*I’m mum alone [single mother] working sixteen hours, my job’s around this area, yeah, if I could live around here it’s fine. All around here, yeah, the shopping close to us, yeah, we are safe around here*” (LF2). Her situation as a single parent in part-time employment underscored the significance of maintaining a stable routine and a sense of safety derived from familiarity with the neighborhood.

However, some participants also pointed out how their local area had changed over time with a growing number of people and cars and a lack of green spaces due to new properties being built. One male participant who moved from London to Bristol stated: “*I don’t like the emissions when they go high, you know, you feel it affects your mental health and your health as well. So, when I come back to Bristol, I always feel better*” (BrM4). Despite acknowledging many benefits of the local area, high urban density neighborhoods such as Brixton in South London made it especially challenging for women to navigate the busy streets with children: “*I had cases where people are just getting very, you know, verbal abusive, where they’re like, oh move, when you have a child with you. Yeah, I feel like the atmosphere is very stressful to be honest, and people, maybe people are stressed*” (LF4).

### Neighbors and everyday social integration

*Xaafad*, or local area, was intertwined with the term *deriska*, translated as ‘neighbors’. As part of an immediate living environment beyond family and household, interactions with neighbors, including non-Somali ones, shaped participants’ experiences of settlement and social integration. All viewed London and Bristol as cities with a diverse mix of cultures and ethnicities. Some participants cherished memories of specific events or extended relationships with ‘English’ or ‘British’ neighbours who welcomed them and helped them settle in their early days in the UK. A twenty-five-year-old participant in North London recalled:

*Neighbors, actually, um, tried to help us settle and there were Somali neighbors and there was also a British lady who was actually giving me some English classes because she felt sorry for me because I wasn’t in school, and I was struggling with language. But she, yeah, it was a lovely experience (BM1).*

During the initial stages of settlement, participants recalled that having ‘British’ or ‘English’ neighbors who could help them with language, food and documentation was particularly important. Some participants used kinship terms to describe their relations with these neighbors. Developing strong ties with neighbors that resemble parental or extended family relations highlighted how personal and formative these experiences have been. For example, one participant in South London described a ‘white English man’ who lived next door and provided support with claiming benefits and asked about his wellbeing and daily activities: “*As a neighbor he was saying like, do you eat, are you okay, how’s your day, did you go to college today, that kind of like, you know, father figure*” (LM4).

Such interactions not only presented opportunities to connect with a diverse group of people but also to reflect on one’s own positionality and identity. Reciprocity with neighbors fostered social integration and a sense of belonging through experiences of cultural exchange. For example, one participant in South London talked about exchanging gifts of food during Eid and Christmas: “*When we’ve got Eid, we give them gifts, like some food with no animal product but just say normal sweets, and at Christmas they brought like a cake, chocolate, stuff like that so we share with each other*” (LF1). Resembling the traditional Somali expression *nabad iyo caano* (peace and milk) which evokes a state of wellbeing, she emphasized the degree of mutual acceptance and reciprocity shared with neighbors: “*Like Somali people, I knocked on the door, ‘can I get a milk?’ if I run out of milk, and she gives me milk, and when she runs out, she knocks my door and I give her milk*” (LF1). This example suggests a strong sense of self-identity and place attachment embedded within the immediate living environment in which neighbors play a key role.

However, neighbors could also be a considerable source of stress for participants who lived in shared accommodation such as hostels or supported housing. They reported witnessing or experiencing incidents involving other residents or outsiders. A participant in Brixton who lived in shared accommodation described how he avoided leaving his room for fear of being racially abused by his neighbor: “*My neighbor, he smoking too much weed and drinking night-time, he not sleeping, sometimes he abuse you, abuse me, yeah, abuse me. He says ‘fucking’, he’s talking to himself, yeah*” (LM1). The transient nature of occupancy in shared accommodation also presented challenges. One female participant in Brixton felt she had little control over her daily living conditions as she had found people from the street inside the premises. She feared for her safety and avoided shared areas such as the kitchen and bathroom. Another participant shared similar experiences, as his sleep was regularly disrupted by anti-social behavior in the shared accommodation: “*A lot of people [in the house], so sometimes I feel dizzy. Because I think it’s downstairs, you know, there are lots of people, sometimes making like you have ‘goo-goo-goo-goo’, like this, I recognise it music*” (LM3). For this reason, these participants tried to spend most of the day outside the accommodation to avoid contact.

Participants’ concerns about their wellbeing also influenced their choices regarding council housing and they expressed reservations about certain neighborhoods due to the fear of encountering racism. As a participant in Bristol explained: “*Our part of Bristol is fine, but only in some area they say that they’re racist. Like Knowle, Whitchurch, that area. Because they get house, but they said we can’t, we are not going because they are scared for their security*” (BrM5). Furthermore, the presence of gang activity was highlighted by many as concerning and distressing. Women especially feared for their safety due to violence, including sexual assaults, and the drugs trade. One participant explained: “*I’ve been kidnapped, I’ve been raped. It’s the same area that I’m living, still I’m living. That’s why I mean, I have a mental problem all that that’s happened to me*” (LF6). She was experiencing flashbacks of the assault and worried about her safety and the safety of her autistic son. Daily encounters with neighbors who made her feel uneasy added to her sense of insecurity. Some male participants who arrived as child refugees recalled conflicts in housing estates that involved threats of violence and altercations. One participant became involved in petty crime and substance use at an early age, he witnessed gang violence and stabbings and believed this experience eventually resulted in a mental health crisis and diagnosis of paranoid schizophrenia. Many participants who were parents therefore considered moving, or wanted to move, to less crime-ridden areas of the city, or even away from the cities if such an opportunity would present itself.

### Life and wellbeing in urban communities

The term ‘community’ was used by participants to refer to both Somali diaspora and social networks and specific places where Somali shops, cafés, madrasas, mosques and other amenities were located. These places served as a socio-cultural infrastructure that facilitated the formation of friendships and close social ties based on everyday interactions. Participants also viewed the community as contributing to the development of local economies, particularly in the Bristol neighborhood of Easton and Willesden in Northwest London, where the Somali diaspora has a prominent presence.

Participants on Stapleton Road in Bristol highlighted the key role played by the Somali community in their neighborhood regeneration due to an increasing number of Somali-owned businesses and their influence on boosting the local economy. A middle-aged male participant stated, for example: “*Before this street was not like this. My friends, they told me before ten years there was nobody here. These shops were closed. But when more Somalian people come in, they open the shops. Because there is good commercial in Somalian people*” (BrM2). Male and female participants said those Somali businesses provided them with employment and an opportunity to cater to their own community. For some it was also an important reason to move to the area. One participant who had moved from Glasgow stated: “*In Glasgow I had no community. At times, I didn’t see anyone to say hello to or look at me in my face. I sell many, many things to people that know me and I’m famous now. Ask anyone, I’m at Somali’s, everyone knows me in Bristol*” (BrM3). Participants recognized the reciprocal relationship between their community, neighborhood and city, mirroring the reciprocal connections they formed with their neighbors. They actively shaped a part of the neighborhood by establishing community spaces and relationships, which in turn contributed to their wellbeing by fostering a sense of belonging and agency. One participant expressed this sentiment, stating: “*When you can see your friends, you get excited, and you get a power from the community*” (BrM3).

Some participants discovered a sense of community beyond the Somali diaspora. They revealed that their mental health conditions compelled them to avoid areas with a high concentration of Somalis because of the intra-community stigma. Instead, they primarily sought support from a close-knit circle of friends, family members and local mental health community groups. A participant in Northwest London described his experience with a local community centre as follows: “*It’s got a good community feeling. They know about my mental health problems, so they always help me in any way they can, which is really encouraging, and yeah, it’s a really nice area*” (BM5). Recently, his condition had significantly improved when he moved back in with his family after he had been sectioned. Being with family helped him find a sense of belonging in the local area and included regular visits to the community centre. Similarly, a female participant in South London shared her experience of being labelled ‘mad’ by her own community, which only worsened her feelings: “*How can you meet somebody who tells you ‘don’t trust, don’t talk to her, don’t listen to her because she’s mad’? That’s just making you worse in your life*” (LF6). While experiencing stigma had reduced her engagement with the Somali community in London, she had discovered a sense of community through her local mental health support group: “*Now they’ve built my life, to be honest, because you have somebody that’s similar. What’s happened to me in the past … but we have somebody who knows your concern or your problems, somebody like you. We just feel like we are family*.”

## Discussion

Urban neighborhoods as human ecological niches can either facilitate or hinder peoples’ ability to lead fulfilling lives. According to this perspective, urban neighborhoods “afford certain ways of acting [ … ] within a particular form of life, as it is lived by a particular ‘kind’ of person” (14:5-7). Instead of solely examining the influence of neighborhood factors on mental health and wellbeing, the three themes emphasize how refugees actively engage in shaping their lives within the urban neighborhoods as eco-social niches. We illustrate that neighborhoods have features that can simultaneously be accommodating, safe and familiar, while also being alien, threatening and unwelcoming. The dynamics of such overlapping neighborhood features are context-dependent, varying across scales and timeframes. For instance, one’s home may feel secure while the surrounding neighborhood may not, or a busy street may induce stress while a café in the local area provides relaxation. Refugees play an active role in shaping their living environment, seeking to maximize their wellbeing and mental health. Their experiences, both positive and negative, are further influenced by factors such as gender, age, and other aspects of their identity that have a significant impact on their mental health and wellbeing.

Research indicates that refugees’ perceptions of their settlement location can evolve over time, presenting therapeutic opportunities under favorable conditions (8, 68, 69). In this study, the participants found a sense of familiarity and safety in their local area, afforded by proximity to essential locations such as markets, shops, workplaces, schools and places of entertainment. Developing a sense of local area contributed to their mental wellbeing. Environmental psychology explains this phenomenon as place attachment, encompassing affective, cognitive and behavioral processes that fulfil basic and psychological needs (70). Because displacement disrupts fundamental human needs, including shelter, security, agency and belonging, it is vital to address and satisfy these needs to develop a sense of place attachment in a post-migration context (71). Recent studies among urban international migrants highlight that meeting place-based needs encourages further exploration and cultivates a sense of home (72). In this context, daily places become meaningful elements of the settlement experience, afforded by physical and social infrastructures (14). Drawing on the perspectives of refugees, our findings contribute the importance of proximate physical environment in this process.

Our findings have shown that within these local areas, Somali refugees actively built community networks. Importantly, such networks were embedded within particular physical locations, such as cafés and shops. It was in these locales that entrepreneurial and cultural spaces were created and formed sources of empowerment and participation in local economies. This supports a growing recognition of the need to provide asylum seekers and refugees with opportunity structures in their respective localities and the importance of social integration and participation (29). While these networks were often co-ethnic and cultural in nature, they also extended beyond the diaspora and as such were essential in serving as a crucial buffer against adverse experiences (73, 74). For example, some participants who disclosed mental health conditions emphasized the importance of local community day centers and mental health charities. They brought up intra-community stigma associated with mental health problems that forced them to distance themselves from their diaspora community members and neighborhood areas (62).

In addition to these communal structures, our study sheds light on the significance of interactions and relationships with neighbors as a facet of social integration that unfolds within urban neighborhood contexts. Interacting with neighbors constituted the most immediate localized form of social contact beyond the household and community networks. This involved reciprocal exchange of household items, gifts and invitations to social gatherings. Being welcomed and accepted by neighbors emerged as a positive indicator of refugee perceived wellbeing from the early days of settlement. Existing research suggests that social inclusion of refugees dispersed to rural areas often hinges on neighborly interactions, where small population size and neighborhood homogeneity make wellbeing both salient and challenging (75). Our study suggests a similar importance of neighborly relations in urban settings, which are typically seen as less socially cohesive and close-knit compared to rural areas (76).

Besides these positive aspects, neighborly relations could also be strained, especially for people living in shared accommodation. Participants living in shared accommodations reported frequently witnessing or experiencing incidents of anti-social behavior and violence, confirming that refugees with psychiatric disorders living in supported housing were exposed to stigma and hostility which has been suggested to cause forced residential mobility (25). At the same time, our study also shows that some participants were unable to move away from places where they felt threatened, either at the level of the local area or within the confines of accommodation. Their choices were constrained either by lack of housing options or by reservations about moving to certain neighborhoods due to fears of racism and discrimination. Some participants, particularly males who arrived as child refugees, recalled conflicts involving violence and criminal activities while growing up. These experiences were aggravated by neighborhood disorder, residential instability and urban density, all of which have been shown to have long-term implications for mental health (51, 77).

Based on our findings, we recommend implementing policies and initiatives that address refugee safety concerns, offer appropriate housing options, and promote positive recreational activities to enhance mental health and wellbeing of refugees within urban neighborhoods. To ensure successful settlement and wellbeing of refugees, it is essential to provide continuous support for education, employment and socialization throughout the entire asylum process and transition into the general welfare system. Enabling refugees to establish their daily lives in familiar and safe environments is crucial for fostering mental health, and minimizing post-migration forced mobility plays a significant role in achieving this. Recognizing the agency of refugees holds the potential to bring about positive transformations in the human ecologies of urban neighborhoods. Community networks should be acknowledged not only as sources of interpersonal support but also as catalysts for local economic development. From an eco-social perspective, the distinction between private and public spaces is not rigid as factors influencing mental health and wellbeing intersect and intertwine across these domains. Rather, as we have shown, the personal and social dimensions are interconnected through the physical and temporal continuity of the living environment.

### Study limitations

The study has several limitations. Firstly, it focused on a specific refugee population, potentially limiting the generalizability of the results to refugee groups with different cultural, ethnic, and socioeconomic backgrounds. Secondly, even within the Somali refugee population, variations exist in terms of social and spatial distribution based on clanship and kinship networks, factors that likely influenced participants’ settlement choices. While this study does not delve into the complexities of Somali clanship, it presents an area for future research. Thirdly, the participants in the study had, on average, resided in the UK for fifteen years, with some having resettled from other European countries. Settlement experiences and perceptions of urban neighborhoods are likely influenced by the trajectory of resettlement and the duration of stay in the country. Nevertheless, the study, benefiting from the longevity of Somali refugee settlement in the UK, offers valuable insights into the enduring influence of refugee life in urban neighborhoods on mental health and wellbeing. The study focused on the post-migration experiences and place as a modifiable risk factor. Therefore, we were not able to address the important role of pre-migration experiences. Lastly, in this study, although some participants disclosed diagnosis of mental health disorders, they were not grouped separately for analysis. However, we have acknowledged relevant mental health diagnoses in our analysis where applicable. While we feel that our predominantly non-clinical sample does not limit the study of place and mental health, future research specifically involving refugees with mental health diagnoses would greatly contribute to our understanding of the influence of place and social ecology in the development and treatment of mental health issues post-migration.

## Conclusion

In this study, we have highlighted how urban neighborhoods, conceived as eco-social niches, influence refugees’ mental health and wellbeing, presenting both challenges and opportunities. The societal and physical features of urban neighborhoods such as significant places near the home, housing environments, interactions with neighbors, and community spaces play an important role in refugee settlement, sense of belonging, and their mental health. By acknowledging the complex and dynamic role of urban surroundings in refugee daily lives, this research emphasizes the primary importance of stable and safe living conditions. This is particularly pertinent given that places where refugees live are a potentially modifiable factor. We also highlight the importance of refugee agency in shaping these environments, with implications for their mental health and wellbeing. Future research could include consideration of broader institutional domains that contribute to the eco-social dynamics of urban environments in which refugees live.

## Data availability statement

The datasets presented in this article are not readily available because the raw qualitative data is not able to be shared since it possesses identifiable information from the participants. Requests to access the datasets should be directed to guntars.ermansons@kcl.ac.uk.

## Ethics statement

The studies involving humans were approved by KCL Research Ethics Committee (HR-19/20-13702); UK Health Research Authority (21/WM/0187). The studies were conducted in accordance with the local legislation and institutional requirements. The participants provided their written informed consent to participate in this study.

## Author contributions

GE: Writing – original draft. HK: Writing – review & editing. PS: Funding acquisition, Writing – review & editing.

## Footnotes

1 While we acknowledge the complexity of this terminology, the 'Global South' refers to countries predominantly in Latin America, Asia, Africa, and Oceania (4). These regions are often distinguished by their historical, socio-economic, and geopolitical characteristics, which typically position them as less economically developed compared to Global North regions such as Europe, North America, and Australia.

2 Since the writing of this article, changes in the application of this policy have resulted in newly recognized refugees having as little as seven days to secure housing and financial support, a task that is virtually impossible (22).

## References

[1] Darling J Bauder H. Introduction: Sanctuary cities and urban struggles: rescaling migration, citizenship, and rights. In: Darling J Bauder H, editors. Sanctuary cities and urban struggles (Manchester: Manchester University Press) (2019). p. 1–22. Available at: https://www.jstor.org/stable/j.ctv18b5jr3.6.

[2] Park H. The power of cities UNHCR Innovation (2016). Available at: https://www.unhcr.org/innovation/the-power-of-cities/ (Accessed March 29, 2023).

[3] World Refugee Council. Refugees and the City: The Twenty-first-century Front Line. Centre for International Governance Innovation (2018). Available online at: https://www.cigionline.org/publications/refugees-and-city-twenty-first-century-front-line/.

[4] Mignolo WD. The global south and world dis/order. J Anthropological Res (2011) 67:165–88. doi: 10.3998/jar.0521004.0067.202

[5] Jacobsen K. Refugees and asylum seekers in urban areas: A livelihoods perspective. J Refugee Stud (2006) 19:273–86. doi: 10.1093/jrs/fel017

[6] Fábos A Kibreab G. Urban refugees: introduction. Refuge: Canada’s J Refugees (2007) 24:3–10. doi: 10.25071/1920-7336.21363

[7] Koizumi K Hoffstaedter G. Urban Refugees: Challenges in Protection, Services and Policy Routledge (2015). doi: 10.4324/9781315733258

[8] Sampson R Gifford SM. Place-making, settlement and well-being: the therapeutic landscapes of recently arrived youth with refugee backgrounds. Health Place (2010) 16:116–31. doi: 10.1016/j.healthplace.2009.09.004 19837625

[9] van Liempt I. From dutch dispersal to ethnic enclaves in the UK: the relationship between segregation and integration examined through the eyes of somalis. Urban Stud (2011) 48:3385–98. doi: 10.1177/0042098010397401

[10] Yashadhana A Alloun E Serova N de Leeuw E Mengesha Z. Place-making and its impact on health and wellbeing among recently resettled refugees in high income contexts: A scoping review. Health Place (2023) 81:103003. doi: 10.1016/j.healthplace.2023.103003 36966669

[11] ONS. Somali individuals in England, Wales and the UK - Office for National Statistics(2023). Available online at: https://www.ons.gov.uk/aboutus/transparencyandgovernance/freedomofinformationfoi/somaliindividualsinenglandwalesandtheuk.

[12] Miller KE Rasmussen A. The mental health of civilians displaced by armed conflict: an ecological model of refugee distress. Epidemiol Psychiatr Sci (2017) 26:129–38. doi: 10.1017/S2045796016000172 PMC6998767 27040595

[13] Kirmayer LJ. Toward an ecosocial psychiatry. World Soc Psychiatry (2019) 1:30. doi: 10.4103/WSP.WSP_9_19

[14] Rose N Birk R Manning N. Towards neuroecosociality: mental health in adversity. Theory Culture Soc (2021) 39:121–144. doi: 10.1177/0263276420981614

[15] Ermansons G Kienzler H Asif Z Schofield P. Refugee mental health and the role of place in the Global North countries: A scoping review. Health Place (2023) 79:102964. doi: 10.1016/j.healthplace.2023.102964 36628805 PMC10265132

[16] Goodfellow M. Hostile Environment: How Immigrants Became Scapegoats London and New York: Verso Books (2020).

[17] Soye E Watters C. Newcomer wellbeing and placemaking in southeast england(2022). Available online at: https://opendocs.ids.ac.uk/opendocs/handle/20.500.12413/17490.

[18] Netto G. Strangers in the city: addressing challenges to the protection, housing and settlement of refugees. Int J Housing Policy (2011) 11:285–303. doi: 10.1080/14616718.2011.599132

[19] Pollard T Howard N. Mental healthcare for asylum-seekers and refugees residing in the United Kingdom: a scoping review of policies, barriers, and enablers. Int J Ment Health Syst (2021) 15:60. doi: 10.1186/s13033-021-00473-z 34127043 PMC8201739

[20] Walsh PW. Asylum and refugee resettlement in the UK. Migration Observatory(2022). Available online at: https://migrationobservatory.ox.ac.uk/resources/briefings/migration-to-the-uk-asylum/.

[21] Hewett A. Living in Limbo: A decade of delays in the UK asylum system (2021). United Kingdom. Available online at: https://www.refugeecouncil.org.uk/wp-content/uploads/2021/07/Living-in-Limbo-A-decade-of-delays-in-the-UK-Asylum-system-July-2021.pdf (Accessed 22 February 2022).

[22] Refugee Council. Thousands of new refugees face destitution and homelessness after being told to leave their accommodation at short notice (2023). Refugee Council. Available online at: https://www.refugeecouncil.org.uk/latest/news/thousands-of-new-refugees-face-destitution-and-homelessness-after-being-told-to-leave-their-accommodation-at-short-notice/ (Accessed December 10, 2023).

[23] Strang AB Baillot H Mignard E. ‘I want to participate.’ transition experiences of new refugees in Glasgow. J Ethnic Migration Stud (2018) 44:197–214. doi: 10.1080/1369183X.2017.1341717

[24] Rowley L Morant N Katona C. Refugees who have experienced extreme cruelty: A qualitative study of mental health and wellbeing after being granted leave to remain in the UK. J Immigrant Refugee Stud (2020) 18:357–74. doi: 10.1080/15562948.2019.1677974

[25] Bhui K Mohamud S Warfa N Curtis S Stansfeld S Craig T. Forced residential mobility and social support: impacts on psychiatric disorders among Somali migrants. BMC Int Health Hum Rights (2012) 12:4. doi: 10.1186/1472-698X-12-4 22510245 PMC3384470

[26] Phillimore J Goodson L. Problem or opportunity? Asylum seekers, refugees, employment and social exclusion in deprived urban areas. Urban Stud (2006) 43:1715–36. doi: 10.1080/00420980600838606

[27] Allsop J Sigona F Phillimore J. ‘Poverty among refugees and asylum seekers in the UK: An evidence and policy review’, IRiS Working Paper Series, No. 1/2014 (Birmingham: Institute for Research into Superdiversity) (2014).

[28] Campbell MR Mann KD Moffatt S Dave M Pearce MS. Social determinants of emotional well-being in new refugees in the UK. Public Health (2018) 164:72–81. doi: 10.1016/j.puhe.2018.07.022 30212722

[29] Phillimore J. Refugee-integration-opportunity structures: shifting the focus from refugees to context. J Refugee Stud (2021) 34:1946–66. doi: 10.1093/jrs/feaa012

[30] Guma T Woods M Yarker S Anderson J. “It’s That Kind of Place Here”: Solidarity, place-making and civil society response to the 2015 refugee crisis in different localities in Wales, UK. Soc Inclusion (2019) 7:96–105. doi: 10.17645/si.v7i2.2002

[31] Biglin J. Photovoice accounts of third places: Refugee and asylum seeker populations’ experiences of therapeutic space. Health Place (2021) 71:102663. doi: 10.1016/j.healthplace.2021.102663 34547566

[32] Biglin J. Embodied and sensory experiences of therapeutic space: Refugee place-making within an urban allotment. Health Place (2020) 62:102309. doi: 10.1016/j.healthplace.2020.102309 32479350

[33] Spicer N. Places of exclusion and inclusion: asylum-seeker and refugee experiences of neighbourhoods in the UK. J Ethnic Migration Stud (2008) 34:491–510. doi: 10.1080/13691830701880350

[34] Mulvey G. In Search of Normality: Refugee Integration in Scotland. Final Report Glasgow: Scottish Refugee Council (2013).

[35] van Liempt I. ‘And then one day they all moved to Leicester’: the relocation of Somalis from the Netherlands to the UK explained. Population Space Place (2011) 17:254–66. doi: 10.1002/psp.605

[36] Shaffer M Stewart E. Refugees on the move: resettlement and onward migration in final destination countries Cheltenham, UK & Northampton, Massachusetts: Handbook of Culture and Migration (2021) p. 341–50.

[37] Schofield P Thygesen M Das-Munshi J Becares L Cantor-Graae E Pedersen C. Ethnic density, urbanicity and psychosis risk for migrant groups – A population cohort study. Schizophr Res (2017) 190:82–7. doi: 10.1016/j.schres.2017.03.032 PMC5735221 28318842

[38] Platts-Fowler D Robinson D. A place for integration: refugee experiences in two english cities. Population Space Place (2015) 21:476–91. doi: 10.1002/psp.1928

[39] Kleist N. Nomads, sailors and refugees: A century of somali migration. Sussex Centre Migration Res United Kingdom: Sussex Centre for Migration Research (2004).

[40] Harris H. The Somali Community in the UK: What we know and how we know it London: Information Centre about Asylum and Refugees (2004).

[41] Valentine G Sporton D Nielsen KB. Identities and belonging: A study of somali refugee and asylum seekers living in the UK and Denmark. Environ Plann D: Soc Space (2009) 27:234–50. doi: 10.1068/d3407

[42] Open Society Foundations. Somalis in London (2014). Open Society Foundations. Available online at: https://www.opensocietyfoundations.org/reports/somalis-london (Accessed February 19, 2018).

[43] Mohdin A. “People were abandoned”: injustices of pandemic laid bare in Brent. In: The Guardian, June 27, sec. UK news (2020). Available at: https://www.theguardian.com/uk-news/2020/jun/27/people-were-abandoned-injustices-of-pandemic-laid-bare-in-brent (Accessed June 27, 2020).

[44] Roig EF. The inner-city neighbourhood with two separate worlds. BristolLive(2021). Available online at: https://www.bristolpost.co.uk/news/bristol-news/life-easton-inner-city-neighbourhood-5919376.

[45] Osman I Samota N Mohamed M Nur A. Somali Community and the state of Employment. Council of Somali Organisations briefing paper June 2015 (London: Council of Somali Organisations) (2015).

[46] GOV.UK. TB incidence and epidemiology in Englan (2021). GOV.UK. Available online at: https://www.gov.uk/government/publications/tuberculosis-in-england-2022-report-data-up-to-end-of-2021/tb-incidence-and-epidemiology-in-england-2021 (Accessed June 14, 2023).

[47] Tulloch A Frayn E Craig TK Nicholson TR. Khat use among Somali mental health service users in South London. Soc Psychiatry Psychiatr Epidemiol (2012) 47:1649–56. doi: 10.1007/s00127-011-0471-8 22249804

[48] Salhi C Scoglio AAJ Ellis H Issa O Lincoln A. The relationship of pre- and post-resettlement violence exposure to mental health among refugees: a multi-site panel survey of somalis in the US and Canada. Soc Psychiatry Psychiatr Epidemiol (2021) 56:1015–23. doi: 10.1007/s00127-020-02010-8 33398495

[49] Lincoln AK Cardeli E Sideridis G Salhi C Miller AB Da Fonseca T. Discrimination, marginalization, belonging, and mental health among Somali immigrants in North America. Am J Orthopsychiatry (2021) 91:280–93. doi: 10.1037/ort0000524 PMC8606194 33289573

[50] Warfa N Curtis S Watters C Carswell K Ingleby D Bhui K. Migration experiences, employment status and psychological distress among Somali immigrants: a mixed-method international study. BMC Public Health (2012) 12:749. doi: 10.1186/1471-2458-12-749 22954304 PMC3489604

[51] Warfa N Bhui K Craig T Curtis S Mohamud S Stansfeld S. Post-migration geographical mobility, mental health and health service utilisation among Somali refugees in the UK: A qualitative study. Health Place (2006) 12:503–15. doi: 10.1016/j.healthplace.2005.08.016 16203171

[52] Bhui K Craig T Mohamud S Warfa N Stansfeld SA Thornicroft G. Mental disorders among Somali refugees. Soc Psychiatry Psychiatr Epidemiol (2006) 41:400–8. doi: 10.1007/s00127-006-0043-5 16520881

[53] Kroll J Yusuf AI Fujiwara K. Psychoses, PTSD, and depression in Somali refugees in Minnesota. Soc Psychiatry Psychiatr Epidemiol (2011) 46:481–93. doi: 10.1007/s00127-010-0216-0 20354676

[54] McCrone P Bhui K Craig T Mohamud S Warfa N Stansfeld SA. Mental health needs, service use and costs among Somali refugees in the UK. Acta Psychiatrica Scandinavica (2005) 111:351–7. doi: 10.1111/j.1600-0447.2004.00494.x 15819728

[55] Ellen Selman L Fox F Aabe N Turner K Rai D Redwood S. ‘You are labelled by your children’s disability’ – A community-based, participatory study of stigma among Somali parents of children with autism living in the United Kingdom. Ethnicity Health (2018) 23:781–96. doi: 10.1080/13557858.2017.1294663 28277014

[56] Linney C Ye S Redwood S Mohamed A Farah A Biddle L. “Crazy person is crazy person. It doesn’t differentiate”: an exploration into Somali views of mental health and access to healthcare in an established UK Somali community. Int J Equity Health (2020) 19:190. doi: 10.1186/s12939-020-01295-0 33109227 PMC7592587

[57] Carroll JK. Murug, waali, and gini: expressions of distress in refugees from Somalia. Primary Care Companion J Clin Psychiatry (2004) 6:119–25. doi: 10.4088/PCC.v06n0303 PMC474735 15361926

[58] Mölsä ME Hjelde KH Tiilikainen M. Changing conceptions of mental distress among somalis in Finland. Transcultural Psychiatry (2010) 47:276–300. doi: 10.1177/1363461510368914 20603389

[59] Kokanovic R Dowrick C Butler E Herrman H Gunn J. Lay accounts of depression amongst Anglo-Australian residents and East African refugees. Soc Sci Med (2008) 66:454–66. doi: 10.1016/j.socscimed.2007.08.019 17923179

[60] Schuchman D McDonald C. Somali Mental Health Vol. 4. Bildhaan: An International Journal of Somali Studies (2008). Available at: https://digitalcommons.macalester.edu/bildhaan/vol4/iss1/8.

[61] Bettmann JE Penney D Freeman PC Lecy N. Somali refugees’ Perceptions of mental illness. Soc Work Health Care (2015) 54:738–57. doi: 10.1080/00981389.2015.1046578 26399492

[62] Ellis BH Lincoln AK Charney ME Ford-Paz R Benson M Strunin L. Mental health service utilization of Somali adolescents: religion, community, and school as gateways to healing. Transcultural Psychiatry (2010) 47:789–811. doi: 10.1177/1363461510379933 21088104

[63] Johnsdotter S Ingvarsdotter K Östman M. Koran reading and negotiation with jinn: strategies to deal with mental ill health among Swedish Somalis. Ment Health Religion Culture (2011) 14:741–55. doi: 10.1080/13674676.2010.521144

[64] Patton MQ. Sampling, qualitative (Purposeful). In: The Blackwell Encyclopedia of Sociology John Wiley & Sons, Ltd (2015).

[65] Hugman R Bartolomei L Pittaway E. Human agency and the meaning of informed consent: reflections on research with refugees. J Refugee Stud (2011) 24:655–71. doi: 10.1093/jrs/fer024

[66] Lewis H. Negotiating anonymity, informed consent and ‘Illegality’: researching forced labour experiences among refugees and asylum seekers in the UK. In: Siegel D de Wildt R, editors. Ethical Concerns in Research on Human Trafficking. Studies of Organized Crime Springer International Publishing, Cham (2016). p. 99–116.

[67] Braun V Clarke V. What can “thematic analysis” offer health and wellbeing researchers? Int J Qual Stud Health Well-being (2014) 9:26152. doi: 10.3402/qhw.v9.26152 25326092 PMC4201665

[68] El-Bialy R Mulay S. Two sides of the same coin: Factors that support and challenge the wellbeing of refugees resettled in a small urban center. Health Place (2015) 35:52–9. doi: 10.1016/j.healthplace.2015.06.009 26203553

[69] Kearns A Whitley E. Getting there? The effects of functional factors, time and place on the social integration of migrants. J Ethnic Migration Stud (2015) 41:2105–29. doi: 10.1080/1369183X.2015.1030374 PMC5412955 28473737

[70] Scannell L Gifford R. Defining place attachment: A tripartite organizing framework. J Environ Psychol (2010) 30:1–10. doi: 10.1016/j.jenvp.2009.09.006

[71] Albers T Ariccio S Weiss LA Dessi F Bonaiuto M. The role of place attachment in promoting refugees’ Well-being and resettlement: A literature review. Int J Environ Res Public Health (2021) 18:11021. doi: 10.3390/ijerph182111021 34769540 PMC8582747

[72] Trąbka A. From functional bonds to place identity: Place attachment of Polish migrants living in London and Oslo. J Environ Psychol (2019) 62:67–73. doi: 10.1016/j.jenvp.2019.02.010

[73] Williams L. Social networks of refugees in the United Kingdom: tradition, tactics and new community spaces. J Ethnic Migration Stud (2006) 32:865–79. doi: 10.1080/13691830600704446

[74] Soller B Goodkind JR Greene RN Browning CR Shantzek C. Ecological networks and community attachment and support among recently resettled refugees. Am J Community Psychol (2018) 61:332–43. doi: 10.1002/ajcp.12240 PMC7592197 29577334

[75] Glorius B Kordel S Weidinger T Bürer M Schneider H Spenger D. Is social contact with the resident population a prerequisite of well-being and place attachment? The case of refugees in rural regions of Germany. Front Sociol (2020) 5:578495. doi: 10.3389/fsoc.2020.578495 33869506 PMC8022740

[76] Avery EE Hermsen JM Kuhl DC. Toward a better understanding of perceptions of neighborhood social cohesion in rural and urban places. Soc Indic Res (2021) 157:523–41. doi: 10.1007/s11205-021-02664-0

[77] Allport T Mace J Farah F Yusuf F Mahdjoubi L Redwood S. ‘Like a life in a cage’: Understanding child play and social interaction in Somali refugee families in the UK. Health Place (2019) 56:191–201. doi: 10.1016/j.healthplace.2019.01.019 30825824

